# Differential Roles of Peroxisome Proliferator-Activated Receptor-*α* and Receptor-*γ* on Renal Crystal Formation in Hyperoxaluric Rodents

**DOI:** 10.1155/2016/9605890

**Published:** 2016-02-28

**Authors:** Kazumi Taguchi, Atsushi Okada, Shuzo Hamamoto, Rei Unno, Takahiro Kobayashi, Ryosuke Ando, Keiichi Tozawa, Bing Gao, Kenjiro Kohri, Takahiro Yasui

**Affiliations:** ^1^Department of Nephrourology, Nagoya City University Graduate School of Medical Sciences, 1 Kawasumi, Mizuho-cho, Mizuho-ku, Nagoya 4678601, Japan; ^2^China-Japan Kidney Stone Research Center, Key Laboratory of Environment and Population Health of Liaoning Education Ministry, Shenyang Medical College, 146 Huanghe North Street, Shenyang 110034, China

## Abstract

Peroxisome proliferator-activated receptors (PPARs) and related inflammatory and oxidative molecule expression were investigated in a hyperoxaluric rodent model to evaluate the* in vivo* efficacy of PPAR agonists in preventing renal crystal formation. PPAR expression was examined in a mouse hyperoxaluria kidney stone model induced by daily intra-abdominal glyoxylate injection. Therapeutic effects of the PPAR*α* agonist fenofibrate and PPAR*γ* agonist pioglitazone were also assessed in a 1% ethylene glycol-induced rat model of hyperoxaluria. Crystal formation, inflammation, cell injury, apoptosis, and oxidative stress were compared to those of vehicle-treated controls. Quantitative reverse transcription-polymerase chain reaction revealed that PPAR*α* and PPAR*γ* expression decrease and increase, respectively, during crystal formation in hyperoxaluric kidneys. In addition, PPAR*α* localized to the cytoplasm of both proximal and distal tubular cells, whereas PPAR*γ* accumulated in the nucleus of proximal tubular cells. Furthermore, renal crystal formation was significantly less prevalent in pioglitazone-treated rats but higher in the fenofibrate-treated and fenofibrate/pioglitazone-cotreated groups compared to controls, thus indicating that pioglitazone, but not fenofibrate, markedly decreased cell inflammation, oxidative stress, and apoptosis. Collectively, the results demonstrated that PPAR*γ* suppressed renal crystal formation via its antioxidative and anti-inflammatory effects; however, the renotoxicity of PPAR*α* may elicit the opposite effect.

## 1. Introduction

Kidney stone disease has lifetime prevalence and recurrence rates nearing 10% and 50%, respectively [1], and is associated with development of end stage renal disease and cardiovascular disease [2, 3]. Recent studies have demonstrated that obesity, inflammation, and oxidative stress-related characteristics similar to metabolic syndrome (MetS) potentiate kidney disease [4]. Moreover, evidence suggests that obesity, weight gain, and high-calorie diets increase the kidney stone formation risk by 1.5–2.0-fold [5]. The estimated annual cost of treatment for kidney stones in the US will rise approximately 1.2-fold to 4.6 billion dollars in 2030 due to the increasing incidence of MetS in the population [6]; thus, a novel preventive therapy is needed to reduce potential burden to the economy and healthcare system.

Kidney stones are hypothesized to form from crystal nidi, which grow, aggregate, and subsequently obstruct the tubular lumen [7]. Numerous studies in hyperoxaluric rat and mouse models induced by ethylene glycol (EG) [8, 9] and glyoxylate (GOX) administration show that renal crystal formation is enhanced by oxidative stress generated in response to ROS and proinflammatory chemokines and cytokines [7, 10]. In addition, the expression of various adipocytokines—such as interleukin-6 (IL-6), monocyte chemotactic protein-1 (MCP-1), and adiponectin (APN)—promotes crystal formation by elevating OPN expression, which associates with the calcium oxalate (CaOx) stone matrix in MetS [11].

Although statins and PPAR agonists are often used to treat MetS-related disease, there is currently no therapy designed to target the molecular mechanism underlying renal crystal formation. PPARs are intranuclear receptors that induce peroxisome proliferation, control gene expression related to hydrocarbons, lipids, and protein metabolism, and facilitate cell differentiation [12]. More specifically, PPAR*α* regulates lipid metabolism by targeting fatty acid and acyl-CoA and thus acts as protective factor in dyslipidemia, whereas PPAR*γ* increases tissue insulin sensitivity and is a pharmaceutical target in diabetes mellitus. Notably, both PPAR*α* and PPAR*γ* agonists reportedly exhibit renoprotective functions through their anti-inflammatory and oxidative effects [13, 14]; however, some studies have published that PPAR agonists show differential effects on oxidative stress and renal function [15, 16].

Therefore, we sought to evaluate the* in vivo* efficacy of PPAR*α*/*γ* agonists in preventing renal crystal formation and investigated the changes in expression of PPARs and other inflammatory and oxidative stress-related molecules in hyperoxaluric animal models.

## 2. Materials and Methods

### 2.1. Animal Procedures

All experimental procedures were performed according to the National Institutes of Health's Guide for the Care and Use of Laboratory Animals, and the procedures were approved by the Animal Care and Use Committee of the Faculty of Medicine, Nagoya City University Graduate School of Medical Sciences.

Male C57BL/6J mice were purchased from Charles River Japan (Yokohama, Japan). The mice were fed with standard chow (MEQ, containing 1.01 g/100 g calcium, 0.78 g/100 g phosphorus, and 0.21 g/100 g magnesium; Oriental Yeast Co., Tokyo, Japan) and had free access to water. Based on our previous report [17], 8-week-old mice were given daily intra-abdominal injections of 80 mg/kg GOX; the kidneys were extracted on days 0, 3, 6, 9, and 12 (*n* = 6 per group) to examine crystal formation and PPAR gene expression.

Eight-week-old male Sprague-Dawley rats (Japan SLC Inc., Shizuoka, Japan) were divided into five groups (*n* = 6/group) to assess the effects of the PPAR*α* and PPAR*γ* agonists, fenofibrate (FF), and pioglitazone (PGZ), respectively. Animals had access to standard chow (CE-2, containing 1.06 g/100 g calcium, 0.99 g/100 g phosphorus, and 0.34 g/100 g magnesium; CLEA Japan Inc., Tokyo, Japan) throughout the experiment. Animals were left untreated (control group) or treated with 1% EG (EG group), 1% EG and 30 mg/kg FF (EG + *α* group), 1% EG and 10 mg/kg PGZ (EG + *γ* group), or 1% EG and 30 mg/kg FF and 10 mg/kg PGZ (EG + *αγ* group). FF and PGZ were administered via the gastric tube as described previously [18, 19]. Blood and 24 h urine samples and kidney sections were collected on day 14 (*n* = 6 per group). Blood and urinary biochemistry were analyzed by LSI Medience Corporation, Tokyo, Japan. Urinary pH and volumes were measured manually. Urinary oxalate concentrations were analyzed using a chemiluminescence analyzer (FOM-110A; Hokuto Denko Corporation, Tokyo, Japan).

### 2.2. Observation of Renal CaOx Crystals

Extracted kidneys were examined for crystal formation by Pizzolato staining and polarized light optical microphotography. Cross sections (4-*μ*m) were stained with Pizzolato according to a previously described method for oxalate-containing crystal detection [20]. Crystal formation was qualitatively assessed in nonstained kidney sections by using polarized light optical microphotography and quantitatively assessed using Image Pro Plus (Media Cybernetics, Inc., Rockville, MD).

### 2.3. Immunohistochemistry of PPARs, Inflammation-Related, and Oxidative Stress-Related Genes

PPAR*α* and PPAR*γ* expression were analyzed by immunohistochemical staining of 4 *μ*m-thick cross sections of mice kidneys. Anti-mouse PPAR*α* rabbit polyclonal antibody (Abcam, Inc., Cambridge, MA) and anti-mouse PPAR*γ* rabbit monoclonal antibody (Cell Signaling Technology, Inc., Danvers, MA) were used as primary antibodies at concentrations of 5 *μ*g/mL and 1.5 *μ*g/mL, respectively. Immunoreactivity was detected using a Histofine Simple Stain Kit for rabbit IgG (Nichirei Biosciences, Inc., Tokyo, Japan).

Immunohistochemistry was carried out for OPN, MCP-1, ED1, APN, and SOD1. Anti-rat OPN rabbit polyclonal antibody (IBL Co., Ltd., Gunma, Japan), anti-rat MCP-1 rabbit polyclonal antibody (Novus Biologicals LLC, Littleton, CO), anti-rat ED1 mouse monoclonal antibody (BMA Biomedicals, Augst, Switzerland), anti-rat APN rabbit polyclonal antibody, and anti-rat SOD1 goat polyclonal antibody (Santa Cruz Biotechnology Inc., Santa Cruz, CA) were used as primary antibodies at concentrations of 20 *μ*g/mL, 10 *μ*g/mL, 10 *μ*g/mL, 1 *μ*g/mL, and 2 *μ*g/mL, respectively. Immunoreactivity was detected using a Histofine Simple Stain Kit for rabbit and mouse IgG (Nichirei Biosciences Inc.) according to the manufacturer's instructions.

### 2.4. Quantitative Reverse Transcription-Polymerase Chain Reaction

Total RNA was extracted from kidney tissues using an RNeasy Midi Kit (Qiagen, Hilden, Germany) according to the manufacturer's instructions. All RNA samples were reverse transcribed into cDNA using a high-capacity cDNA Reverse Transcription Kit (Applied Biosystems, Foster City, CA). TaqMan gene expression assays and FAM dye-labeled TaqMan MGB probes were obtained with the following mRNA sequences:* Spp1* (Rn00681031, encoding OPN),* Ccl2* (Rn00580555, encoding MCP-1),* Cd68* (Rn01495634, encoding ED1),* Adipoq* (Rn00595250_m1, encoding APN),* Sod1* (Rn00566938, encoding SOD1),* Ppara* (Rn00566193_m1 and Mm00440939_m1, encoding PPAR*α*), and* Pparg* (Rn00440945_m1 and Mm01184322_m1, encoding PPAR*γ*). Reactions were performed using TaqMan® Fast Universal PCR Master Mix (4352042; Applied Biosystems) and a 7500 Fast Real-Time PCR System (Applied Biosystems). The expression of each gene was normalized to that of a *β*-actin internal control (*Actb*; Mm00607939_s1, or Rn00667869_m1). The corrected expression for each sample was normalized to the average value on day 0 (for mouse study) or treatment controls (for rat study).

### 2.5. Western Blotting

Whole-protein extracts prepared by sonication were separated on 12.5% sodium dodecyl sulfate polyacrylamide gels (SDS-PAGE) and transferred to immobilon-H polyvinylidene difluoride (PVDF) membranes (Millipore). After blocking with Tris-buffered saline (pH 7.5) Tween 20 containing 5% skimmed milk, the membranes were incubated with anti-PPAR*α* rabbit antibody (Abcam), anti-PPAR*γ* rabbit antibody (Cell Signaling Technology), anti-caspase-3 rabbit antibody (Cell Signaling Technology), and anti-caspase-9 mouse antibody (Cell Signaling Technology) followed by horseradish peroxidase-conjugated secondary antibody (GE Healthcare, Piscataway, NJ, USA). Chemiluminescent signals were visualized using ECL Western blotting detection reagents and scanned using a LAS4000 analyzer (GE Healthcare).

### 2.6. Evaluation of Apoptosis

TUNEL assays were performed to detect apoptotic renal tubular cells by using an in situ cell death detection kit (Roche Applied Science, Indianapolis, IN). The number of TUNEL-positive cells was counted in 20 different fields for each section under ×400 magnification.

### 2.7. Statistical Analysis

All data are expressed as mean ± standard error. The statistical analyses were performed using a two-way ANOVA for comparisons among three or more groups, or the Mann-Whitney *U* test for comparisons between two groups. All statistical analyses were performed using Statistical Analysis System, version 9.1 (SAS Institute Inc., Cary, NC). *p* values < 0.05 were considered statistically significant.

## 3. Results

### 3.1. Renal CaOx Crystal Deposits and PPAR Expression in Hyperoxaluric Mice

**Figure 1:**
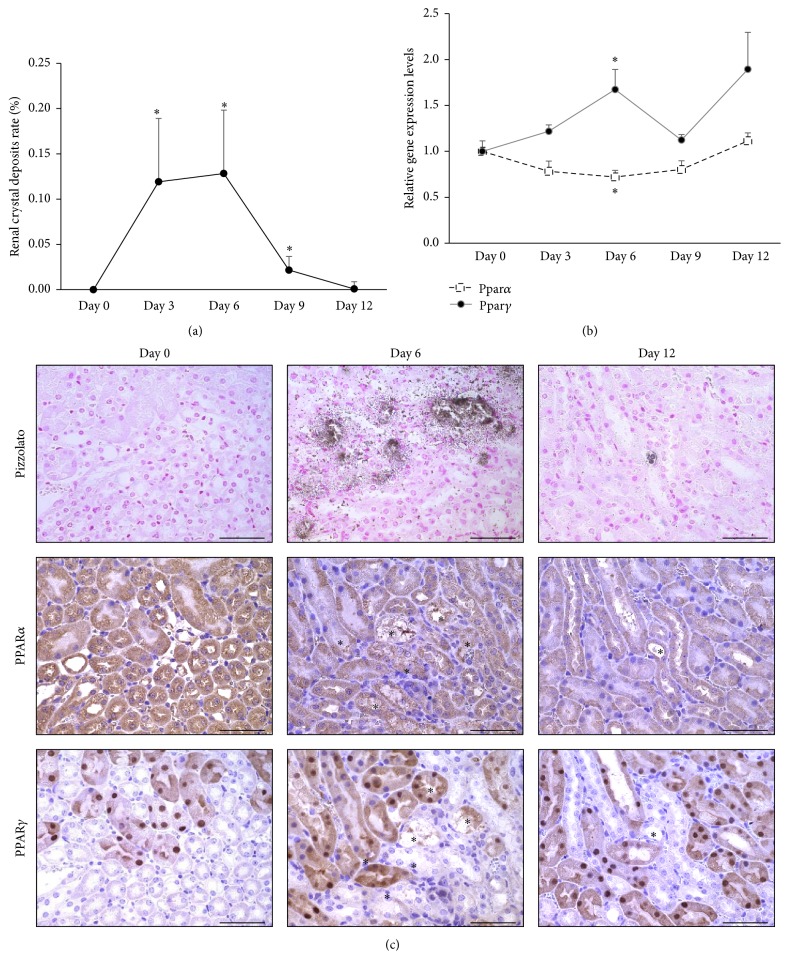
Renal CaOx crystal formation and PPAR expression in hyperoxaluric mice. (a) Quantitative estimation of renal CaOx crystals. Crystallization in each kidney section on designed time points was quantified by calculating the ratio (%) of the area containing crystals to that of the entire kidney section by using Image Pro Plus. (b) Evaluation of PPAR*α* and PPAR*γ* expression by qRT-PCR using TaqMan assays (*n* = 6 for each time point). Control values represent the average on day 0 and data are presented as mean ± SE. ^*∗*^ *p* < 0.05 versus gene expression on day 0. (c) PPAR*α* and PPAR*γ* immunohistochemistry in hyperoxaluric mouse kidneys obtained on days 0, 6, and 12. Each asterisk represents the location of CaOx crystals detected by Pizzolato staining. Magnification: 400x. Scale bar = 50 *μ*m.

To clarify the roles of PPAR*α* and PPAR*γ* in kidney stone formation, we first examined their expression using hyperoxaluric stone model mice. Renal crystal deposits were detected after 3 days of GOX administration, peaked on day 6, and disappeared by day 12 (Figure 1(a)), consistent with previous reports [12]. PPAR*γ* mRNA expression analysis in renal tissue from GOX-treated mice showed an increase in transcript expression on day 6, which is in direct contrast to the lower PPAR*α* observed on day 6 (Figure 1(b)).

Moreover, CaOx deposits detected by Pizzolato staining were localized to the intratubular space at the corticomedullary junction of mouse kidneys. Strong PPAR*α* staining was observed in the cytoplasm of both proximal and distal tubules, particularly in day 0 controls, whereas PPAR*γ* was detected in nucleus of proximal tubular cells, but not in the tubular cells affected by CaOx crystals (Figure 1(c)).

### 3.2. Observation of Renal CaOx Crystals in Hyperoxaluric Rats Treated with PPAR Agonists

**Figure 2:**
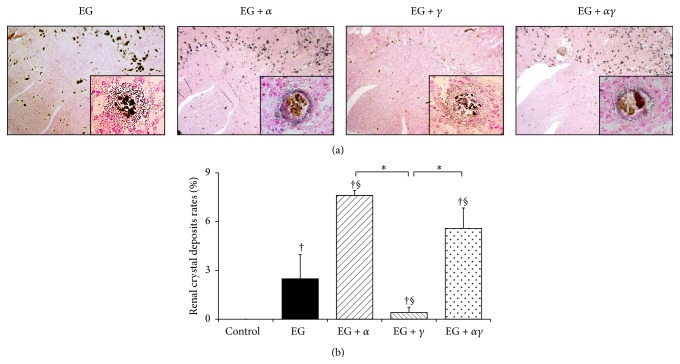
Deposits of renal CaOx crystals in model rats observed using Pizzolato staining and quantitative estimation. (a) Representative micrographs of renal sections obtained on day 14. Magnification: 40x (inset: 400x). (b) Quantitative estimation of renal CaOx crystals. Crystallization in each kidney section was quantified by calculating the ratio (%) of the area with crystals to that of the entire kidney section by using Image Pro Plus (*n* = 6 for all groups). Data are presented as mean ± SE. ^*∗*^ *p* < 0.05 among EG + *α*, EG + *γ*, and EG + *αγ* groups; ^†^ *p* < 0.05 compared with the control group; ^§^ *p* < 0.05 compared with the EG group. Data of control, EG, and EG + *γ* groups obtained by our previous study [26].

Intratubular CaOx crystal deposits developed in the corticomedullary lesion of rat kidneys after 14 days of EG administration, as detected by Pizzolato staining (Figure 2(a)). Notably, larger renal crystal deposits were observed in the EG + *α* and EG + *αγ* groups and smaller amounts were found in the EG + *γ* group than in the EG and control groups. No significant difference in crystal deposits rates was found between the EG + *α* and EG + *αγ* groups (Figure 2(b)).

### 3.3. Physical, Serum, and Urinary Variables

The body weight of the EG + *α* and EG + *αγ* groups was significantly lower than that in the control and EG groups, while body weight of the EG + *γ* group was lower than that of the control group but did not differ from that of the EG group. In addition, kidney weights in the EG, EG + *α*, and EG + *αγ* groups were markedly higher than that of controls, whereas these were significantly lower in the EG + *γ* group than in the EG group (Table 1).

In terms of serum biochemistry, creatinine and phosphorus levels were significantly higher in EG + *α* and EG + *αγ* rats than in their control and EG counterparts. No statistical differences were found among the five groups with respect to serum calcium or triglyceride levels, except for the EG group. Furthermore, serum blood sugar concentrations were lower in the EG + *γ* and EG + *αγ* groups than in others, while total cholesterol concentrations were lower in the EG + *α* and EG + *αγ* groups. (Table 1)

Urinary volume and oxalate excretion were markedly higher in all EG-treated groups, whereas calcium excretion was lower than that in the control group. No statistical differences were found in urinary pH or phosphorus excretion across all treatment groups (Table 1).

### 3.4. PPAR*α* and PPAR*γ* Expression in Hyperoxaluric Rats

**Figure 3:**
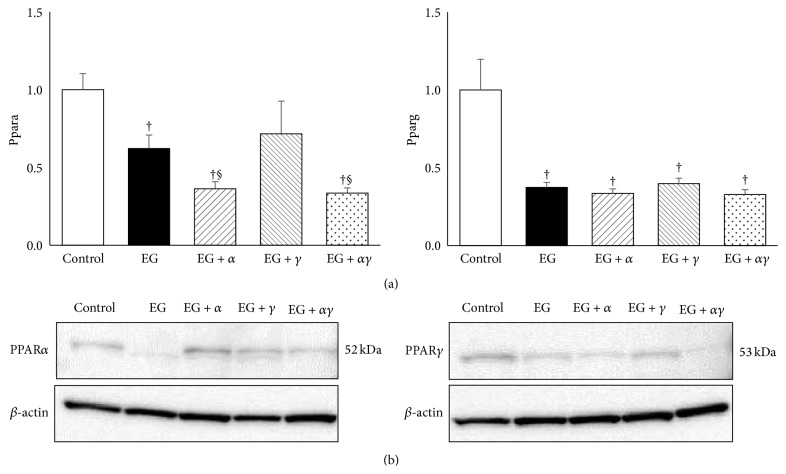
PPAR*α* and PPAR*γ* gene expression in model rat kidneys. (a) Gene expression was assessed by qRT-PCR using TaqMan assays (*n* = 6 for all groups). Data are presented as mean ± SE. ^†^ *p* < 0.05 compared with the control group; ^§^ *p* < 0.05 compared to the EG group. (b) Western blotting of rat kidneys for PPAR*α* and PPAR*γ* protein expression. The molecular mass of PPAR*α* and PPAR*γ* was confirmed by bands at 52 and 53 kDa, respectively.

PPAR*α* mRNA expression was lower in the EG, EG + *α*, and EG + *γ* groups than that in the control group. Particularly, mRNA expression of PPAR*α* was the lowest in the EG + *α* and EG + *αγ*. PPAR*γ* mRNA expression was lower in all EG-treated groups than that in the control group; however, there were no significant differences among them (Figure 3(a)).

On the contrary, PPAR*α* and PPAR*γ* protein expression were higher in the EG + *α* and EG + *γ* groups when compared with the other groups, respectively (Figure 3(b)).

### 3.5. Proinflammatory-Related Gene Expressions

**Figure 4:**
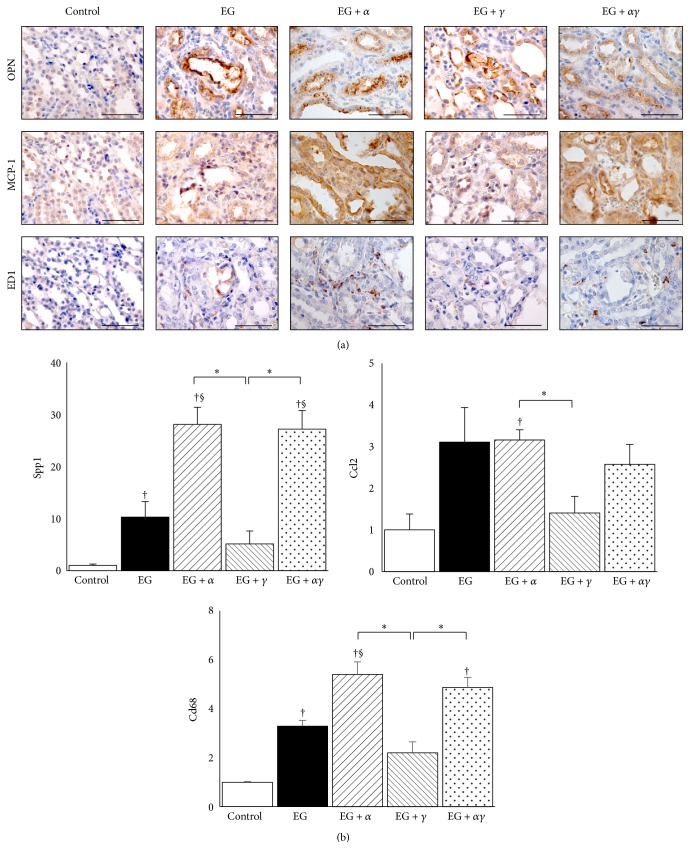
Proinflammatory gene expression in model rat kidneys. (a) Distribution of proinflammatory-related gene expression in rat kidneys obtained on day 14. OPN, osteopontin; MCP-1, monocyte chemoattractant protein-1. Magnification: 400x. Scale bar = 50 *μ*m. (b) Gene expression was assessed by qRT-PCR using TaqMan assays (*n* = 6 for all groups). Data are presented as mean ± SE. ^*∗*^ *p* < 0.05 among EG + *α*, EG + *γ*, and EG + *αγ* groups; ^†^ *p* < 0.05 compared with the control group; ^§^ *p* < 0.05 compared to the EG group.

The macrophage proinflammatory proteins OPN, MCP-1, and ED1 were strongly detected in the corticomedullary region of EG, EG + *α*, and EG + *αγ* rat kidneys (Figure 4(a)). OPN was observed in inner tubule side of renal proximal tubular cells, whereas MCP-1 was observed in intracellular space of both renal tubular and interstitial cells. ED1-positive cells localized to the peritubular and interstitial space around crystal deposits. In the EG + *γ* group, MCP-1 and ED1 expression was weak when compared to the EG, EG + *α*, and EG + *αγ* groups.

In qRT-PCR analyses,* Spp1* and* Cd68* were highly expressed in the EG, EG + *α*, and EG + *αγ* groups than in the control group, with expression in the EG + *α* group notably higher than that in the EG and EG + *γ* groups.* Ccl2* expression in the EG + *α* group was markedly higher than that in the control and EG + *γ* groups (Figure 4(b)).

### 3.6. Anti-Inflammatory and Oxidative Stress Gene Expression

**Figure 5:**
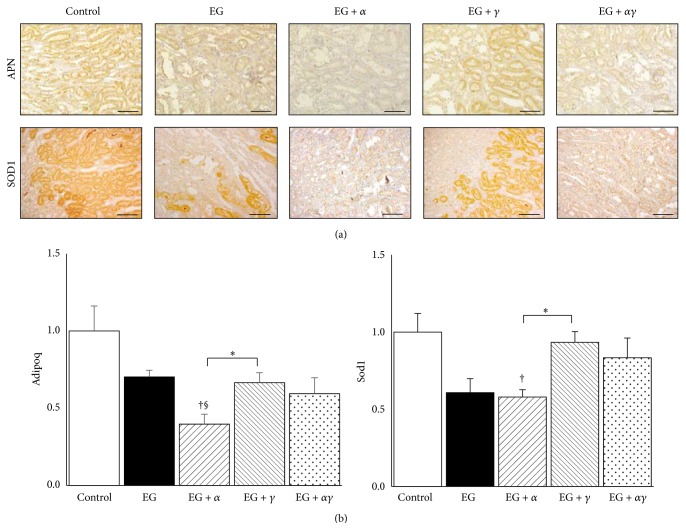
Anti-inflammatory and oxidative stress gene expression in model rat kidneys. (a) Distribution of anti-inflammatory and oxidative stress-related gene expressions in rat kidneys obtained on day 14. APN, adiponectin; SOD1, superoxide dismutase 1. Magnification: 200x. Scale bar = 100 *μ*m. (b) Gene expression was measured by qRT-PCR using TaqMan assays (*n* = 6 for all groups). Data are presented as the mean ± SE. ^*∗*^ *p* < 0.05 among EG + *α*, EG + *γ*, and EG + *αγ* groups; ^†^ *p* < 0.05 compared with the control group; ^§^ *p* < 0.05 compared to the EG group.

APN and SOD1 immunostaining demonstrated reduced expression in the EG, EG + *α*, and EG + *αγ* groups than in the control and EG + *γ* groups, which localized to renal tubular cells in the corticomedullary and medullary space (Figure 5(a)).

By qRT-PCR, Adipoq and Sod1 expression in the EG + *α* group were significantly lower than those in the control and EG + *γ* groups. Adipoq expression in the EG + *α* group was also lower than that in the EG group (Figure 5(b)).

### 3.7. Evaluation of Cellular Apoptosis

**Figure 6:**
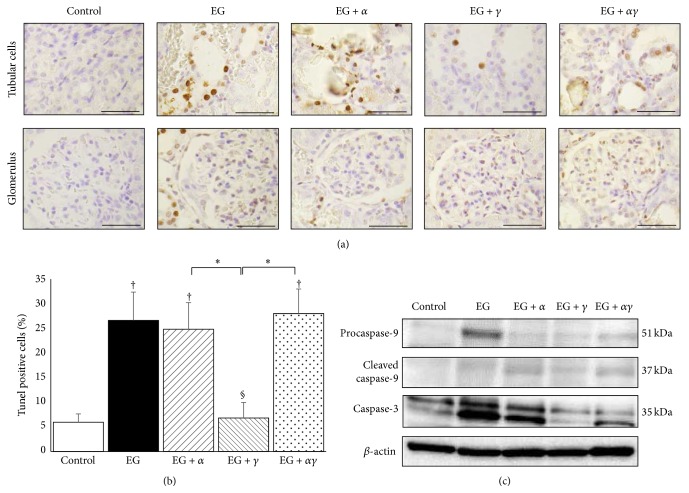
Evaluation for apoptotic cells in model rat kidneys. (a) Representative micrographs of TUNEL-stained renal sections obtained on day 14. Magnification: 400x. Scale bar = 50 *μ*m. (b) The average number of TUNEL-positive cells per high power field (400x; *n* = 20 fields per group). Data are presented as mean ± SE. ^*∗*^ *p* < 0.05 among EG + *α*, EG + *γ*, and EG + *αγ* groups; ^†^ *p* < 0.05 compared to the control group; ^§^ *p* < 0.05 compared to the EG group. (c) Western blotting of rat kidneys for caspase-9 and caspase-3 protein levels. The molecular mass of procaspase-9, cleaved caspase-9, and caspase-3 was confirmed by bands at 51, 37, and 35 kDa, respectively.

TUNEL-positive cells were observed within the renal proximal tubule mostly localized around crystal deposits and the glomerulus in EG-treated kidneys (Figure 6(a)). The number of TUNEL-positive cells in the EG, EG + *α*, and EG + *αγ* groups was significantly higher than in the control and EG + *γ* groups (Figure 6(b)).

Procaspase-9 expression was high in the EG and EG + *αγ* groups, whereas cleaved caspase-9 cleavage fragments were highly detected in the EG + *α* and EG + *αγ* when compared to other groups. In addition, caspase-3 expression was markedly higher in the EG, EG + *α*, and EG + *αγ* groups than the other groups (Figure 6(c)).

## 4. Discussion

PPARs are transcription factors that regulate gene expression related to effects of intracellular lipids and inflammatory mediators. Although the exact role of PPARs in kidney stone pathogenesis is yet unknown, we revealed that PPAR*α* and PPAR*γ* exhibit differential effects on this process in hyperoxaluric mice and rats. More specifically, GOX-treated mice showed renal CaOx crystal depositions on days 3 and 6, which decreased by day 9. Interestingly, the expression of these PPARs was different—with lower PPAR*α* expression and higher PPAR*γ* expression during renal crystal formation. Additionally, PPAR*α* localized within the tubular cytoplasm among proximal and distal tubules and collecting ducts, whereas PPAR*γ* was observed in the nucleus of proximal tubules as previously reported [21], but not in crystal-induced injured tubules. These findings are indicative of sufficient, and possibly heightened, PPAR*α* expression in renal tubular cells; however, PPAR*γ* expression was specifically found in the tubules in the cortex under a normal physiological state. Moreover, CaOx crystal development decreased PPAR*α* expression throughout the kidney but increased PPAR*γ* expression in healthy tubular cells that were undamaged by crystal deposits.

Based on results from the experimental rat model, we identified that the two PPAR agonists have differential effects on kidney stone formation: PGZ and FF attenuated and accelerated EG-induced crystal development, respectively. With regard to urinary mineral excretion, no significant differences were observed among EG, EG + *α*, EG + *γ*, and EG + *αγ* groups. Since rats generally prefer to drink EG over water, all EG-treated groups had higher urine volume compared to the control group. Moreover, the urinary calcium excretions in all EG-treated rats were lower than those in controls because of the formation of renal CaOx crystals [22]. These results support known evidence that PGZ and FF do not interfere with urinary parameters that affect renal crystal development.

PPAR*γ* agonists, such as PGZ, have anti-inflammatory and antioxidative effects. PGZ treatment suppressed inflammatory-related gene expression including that of OPN, MCP-1, and ED1, the number of TUNEL-positive apoptotic cells, and caspase-9 and caspase-3 expression, whereas these were increased in FF-treated rats. FF rats showed decreased APN and Sod1 expression, which are considered anti-inflammatory and antioxidative molecules. Kidney stone formation results from reactive oxygen species (ROS) production, which is facilitated by inflammatory molecules and macrophage infiltration [23]. Consistently, metabolic disorders accelerate the stone development via inflammatory adipocytokines, which is hindered by APN expression [24, 25].

Some reports, including our previous study, have suggested that PGZ suppressed renal crystal formation as well as acute renal injury by inhibiting cellular inflammation, ROS production, and apoptosis in rat models [13, 26]. Mechanistically, FF binds to and activates PPAR*α*, thereby reducing fasting triglyceride and low-density cholesterol levels in blood plasma. In addition, Frazier-Wood and colleagues reported that FF treatment increased serum MCP-1 but decreased serum APN concentrations during a single nucleotide polymorphism study of human PPAR*α* [27].

In this study, treatment with PPAR agonist suppressed body weight gain in rats. EG and FF treatment enlarged the rat's kidney, and FF increased the serum level of creatinine, which is likely the result of FF-induced renotoxicity, particularly during the early phrase of treatment. Patients with older age, of male gender, with high serum creatinine, and using high doses of FF are considered at high risk for adverse effects. FF is hypothesized to reduce the renal blood flow by affecting the expression of vasodilatory prostaglandins [28]. Stoller and colleagues insisted that kidney stone development is caused by renovascular flow dysfunction [29]. We used 30 mg·kg^−1^·day^−1^ of FF for rat, which was 10 times higher than the human dose of 3 mg·kg^−1^·day^−1^. Besides, these “male” rats already had the relative serum creatinine elevation and crystal-induced tubular cell injury. As such, the adverse effects of high-dose FF and crystal-induced renotoxicity may further accelerate crystal deposit formation and renal dysfunction.

Despite promising results, our study is limited by the dissimilarities between the various animal models of kidney stone development. It has two primary limitations. For instance, rodent stone models develop acute and excessive renal crystal deposits compared to humans; however, similarities between the mechanisms of intratubular crystal formation make these models suitable for translational research on renal disease. Additionally, the doses of PPAR agonists given to rats in our study were relatively higher than those used in humans; however, our results on PPAR*α* and PPAR*γ* renal expression showed that appropriate drug delivery should be investigated further. Further pharmacokinetics studies are needed to clarify the significance of our results.

## 5. Conclusions

Collectively, we established the differential effects of PPAR agonists on renal crystal formation, where PPAR*α* and PPAR*γ* convey renotoxic and renoprotective effects, respectively, in the hyperoxaluric kidney environment. Since PPAR*α* and PPAR*γ* are differentially expressed in the kidneys, it is reasonable that these two effectors play opposing roles in tubular cell CaOx crystal deposition and should be considered when treating MetS patients with kidney stones.

## Figures and Tables

**Table 1:** Physical characteristics and serum and urinary biochemistry of experimental rats on day 14.

Physical findings	Control	EG	EG + *α*	EG + *γ*	EG + *αγ*
Body weight gain (g)	92.0 ± 4.6	81.0 ± 4.0	53.8 ± 3.4^†§^	66.8 ± 3.1^†^	63.2 ± 2.7^†§^
Kidney weight (g)	2.3 ± 0.1	3.3 ± 0.4^†^	4.4 ± 0.1^†§^	2.5 ± 0.1^§^	4.4 ± 0.2^†§^

Serum biochemistries	Control	EG	EG + *α*	EG + *γ*	EG + *αγ*

Cr (mg/dL)	0.25 ± 0.01	0.30 ± 0.03	0.44 ± 0.03^†§^	0.28 ± 0.02	0.47 ± 0.04^†§^
Ca (mg/dL)	10.2 ± 0.1	10.7 ± 0.1	10.2 ± 0.1	10.4 ± 0.2	10.2 ± 0.1
P (mg/dL)	8.4 ± 0.5	8.0 ± 0.1	9.9 ± 0.3^†§^	7.6 ± 0.1	8.8 ± 0.1^†§^
BS (mg/dL)	146 ± 6	148 ± 2	142 ± 6	123 ± 7^†§^	127 ± 3^†§^
TG (mg/dL)	36 ± 2	41 ± 10^†^	39 ± 9	47 ± 8	43 ± 9
TC (mg/dL)	56 ± 2	54 ± 2	45 ± 3^†^	52 ± 2	33 ± 3^†§^

Urine biochemistries	Control	EG	EG + *α*	EG + *γ*	EG + *αγ*

Volume (mL)	11 ± 1	29 ± 5^†^	28 ± 2^†^	24 ± 3^†^	21 ± 3^†^
pH	7.8 ± 0.1	7.6 ± 0.3	7.3 ± 0.2	7.6 ± 0.2	7.3 ± 0.3
Ca (nmol/g/Cr)	86 ± 11	43 ± 6^†^	43 ± 3^†^	43 ± 7^†^	31 ± 6^†^
P (*µ*mol/g/Cr)	2.6 ± 0.3	2.6 ± 0.7	2.8 ± 0.7	2.7 ± 0.2	2.6 ± 0.3
Ox (*µ*mol/g/Cr)	24 ± 3	201 ± 27^†^	343 ± 40^†§^	230 ± 37^†^	229 ± 30^†^

Mean ± standard error (SE).

^†^ *p* < 0.05 compared with the control group; ^§^ *p* < 0.05 compared with the EG group. Data of control, EG, and EG + *γ* groups were obtained by our previous study [26].

Cr, creatinine; Ca, calcium; P, phosphorus; BS, blood sugar; TG, triglyceride; TC, total cholesterol; Ox, oxalate.

## References

[1] Neisius A., Preminger G. M. (2013). Stones in 2012: epidemiology, prevention and redefining therapeutic standards. *Nature Reviews Urology*.

[2] El-Zoghby Z. M., Lieske J. C., Foley R. N. (2012). Urolithiasis and the risk of ESRD. *Clinical Journal of the American Society of Nephrology*.

[3] Ando R., Nagaya T., Suzuki S. (2013). Kidney stone formation is positively associated with conventional risk factors for coronary heart disease in Japanese men. *The Journal of Urology*.

[4] Kohjimoto Y., Sasaki Y., Iguchi M., Matsumura N., Inagaki T., Hara I. (2013). Association of metabolic syndrome traits and severity of kidney stones: results from a nationwide survey on urolithiasis in Japan. *American Journal of Kidney Diseases*.

[5] Lieske J. C. (2014). New insights regarding the interrelationship of obesity, diet, physical activity, and kidney stones. *Journal of the American Society of Nephrology*.

[6] Antonelli J. A., Maalouf N. M., Pearle M. S., Lotan Y. (2014). Use of the National Health and Nutrition Examination Survey to calculate the impact of obesity and diabetes on cost and prevalence of urolithiasis in 2030. *European Urology*.

[7] Okada A., Yasui T., Hamamoto S. (2009). Genome-wide analysis of genes related to kidney stone formation and elimination in the calcium oxalate nephrolithiasis model mouse: detection of stone-preventive factors and involvement of macrophage activity. *Journal of Bone and Mineral Research*.

[8] Khan S. R., Glenton P. A., Byer K. J. (2006). Modeling of hyperoxaluric calcium oxalate nephrolithiasis: experimental induction of hyperoxaluria by hydroxy-L-proline. *Kidney International*.

[9] Joshi S., Wang W., Peck A. B., Khan S. R. (2015). Activation of the NLRP3 inflammasome in association with calcium oxalate crystal induced reactive oxygen species in kidneys. *The Journal of Urology*.

[10] Taguchi K., Okada A., Kitamura H. (2014). Colony-stimulating factor-1 signaling suppresses renal crystal formation. *Journal of the American Society of Nephrology*.

[11] Zuo L., Tozawa K., Okada A. (2014). A paracrine mechanism involving renal tubular cells, adipocytes and macrophages promotes kidney stone formation in a simulated metabolic syndrome environment. *The Journal of Urology*.

[12] Michalik L., Auwerx J., Berger J. P. (2006). International union of pharmacology. LXI. Peroxisome proliferator-activated receptors. *Pharmacological Reviews*.

[13] Reel B., Guzeloglu M., Bagriyanik A. (2013). The effects of PPAR-*γ* agonist pioglitazone on renal ischemia/reperfusion injury in rats. *Journal of Surgical Research*.

[14] Zhou Y., Kong X., Zhao P. (2011). Peroxisome proliferator-activated receptor-*α* is renoprotective in doxorubicin-induced glomerular injury. *Kidney International*.

[15] Ruilope L., Hanefeld M., Lincoff A. (2014). Effects of the dual peroxisome proliferator-activated receptor-*α*/*γ* agonist aleglitazar on renal function in patients with stage 3 chronic kidney disease and type 2 diabetes: a phase IIb, randomized study. *BMC Nephrology*.

[16] El-Sheikh A. A. K., Rifaai R. A. (2014). Peroxisome proliferator activator receptor (PPAR)-*γ* ligand, but not PPAR-*α*, ameliorates cyclophosphamide-induced oxidative stress and inflammation in rat liver. *PPAR Research*.

[17] Okada A., Nomura S., Saeki Y. (2008). Morphological conversion of calcium oxalate crystals into stones is regulated by osteopontin in mouse kidney. *Journal of Bone and Mineral Research*.

[18] Kadian S., Mahadevan N., Balakumar P. (2013). Differential effects of low-dose fenofibrate treatment in diabetic rats with early onset nephropathy and established nephropathy. *European Journal of Pharmacology*.

[19] Yang H.-C., Deleuze S., Zuo Y., Potthoff S. A., Ma L.-J., Fogo A. B. (2009). The PPAR*γ* agonist pioglitazone ameliorates aging-related progressive renal injury. *Journal of the American Society of Nephrology*.

[20] Pizzolato P. (1964). Histochemical recognition of calcium oxalate. *Journal of Histochemistry & Cytochemistry*.

[21] Li S., Nagothu K. K., Desai V. (2009). Transgenic expression of proximal tubule peroxisome proliferator-activated receptor-*α* in mice confers protection during acute kidney injury. *Kidney International*.

[22] Li Y., McMartin K. E. (2009). Strain differences in urinary factors that promote calcium oxalate crystal formation in the kidneys of ethylene glycol-treated rats. *The American Journal of Physiology—Renal Physiology*.

[23] Khan S. R. (2013). Reactive oxygen species as the molecular modulators of calcium oxalate kidney stone formation: evidence from clinical and experimental investigations. *The Journal of Urology*.

[24] Taguchi K., Okada A., Hamamoto S. (2015). Proinflammatory and metabolic changes facilitate renal crystal deposition in an obese mouse model of metabolic syndrome. *The Journal of Urology*.

[25] Fujii Y., Okada A., Yasui T. (2013). Effect of adiponectin on kidney crystal formation in metabolic syndrome model mice via inhibition of inflammation and apoptosis. *PLoS ONE*.

[26] Taguchi K., Okada A., Yasui T. (2012). Pioglitazone, a peroxisome proliferator activated receptor *γ* agonist, decreases renal crystal deposition, oxidative stress and inflammation in hyperoxaluric rats. *Journal of Urology*.

[27] Frazier-Wood A. C., Ordovas J. M., Straka R. J. (2013). The PPAR alpha gene is associated with triglyceride, low-density cholesterol and inflammation marker response to fenofibrate intervention: the GOLDN study. *Pharmacogenomics Journal*.

[28] Kostapanos M. S., Florentin M., Elisaf M. S. (2013). Fenofibrate and the kidney: an overview. *European Journal of Clinical Investigation*.

[29] Stoller M. L., Meng M. V., Abrahams H. M., Kane J. P. (2004). The primary stone event: a new hypothesis involving a vascular etiology. *Journal of Urology*.

